# Dual Approach for Basilar Artery Fenestration Aneurysm With Insights From Computational Fluid Dynamics

**DOI:** 10.1227/neuprac.0000000000000122

**Published:** 2024-11-11

**Authors:** Rafael T. Tatit, Vincenzo T. R. Loly, Rabih G. Tawk, Johnny S. Sandhu, Lucas O. C. Guida, Jorge Rios-Zermeno, João S. B. Lima, Carlos E. Baccin

**Affiliations:** * Department of Interventional Neuroradiology, Hospital Beneficência Portuguesa de São Paulo, São Paulo, Brazil;; ‡ Department of Neurosurgery, Mayo Clinic, Jacksonville, Florida, USA;; § Department of Radiology, Mayo Clinic, Jacksonville, Florida, USA;; ‖ Department of Mechanical Engineering, Instituto Mauá de Tecnologia, São Paulo, Brazil

**Keywords:** Aneurysm, Basilar artery fenestration, Computational fluid dynamics, Endovascular intervention, Hemodynamics, Wall shear stress

## Abstract

**BACKGROUND AND IMPORTANCE:**

Basilar artery fenestration aneurysms (BAFAs) present significant clinical challenges because of their high rupture risk and complex anatomy. Comprehensive management strategies are required, including thorough vascular assessment and post-treatment surveillance. Integration of complementary analyses such as computational fluid dynamics (CFD) holds promise in facilitating preoperative planning for these intricate lesions.

**CLINICAL PRESENTATION:**

A 60-year-old female was diagnosed with a BAFA during evaluation for headaches. Digital subtraction angiography revealed the aneurysm projecting posteriorly with a neck predominantly based on the right limb of the fenestration. After initial treatment with a pipeline embolization device, the aneurysm remained patent, requiring further treatment. A secondary approach with stent-assisted loose-packing coil embolization was then performed, resulting in complete occlusion of the aneurysm. Analysis of pretreatment 3-dimensional rotational angiogram images with CFD provided critical hemodynamic insights. It identified that 38.89% of the aneurysm area was exposed to low wall shear stress (WSS) and 11.5% was exposed to high WSS, indicating a high rupture risk profile with significant areas of both low and high WSS. In addition, low WSS was observed in regions corresponding to daughter sacs, suggesting a higher rupture risk. Streamline analysis indicated increased inflow through the right vertebral artery and the right limb of the fenestration, supporting the initial angiographic impression and guiding the choice of therapeutic strategy.

**CONCLUSION:**

This is the first study to analyze the morphology and pretreatment hemodynamics of a BAFA using CFD, illustrating the potential for future development of individualized therapeutic approach–based CFD in complex aneurysms.

**ABBREVIATIONS:**
BAFA: basilar artery fenestration aneurysm
CFD: computational fluid dynamics
PED: pipeline embolization device
WSS: wall shear stress.

Basilar artery fenestration aneurysms (BAFAs) represent rare vascular anomalies that typically arise in the proximal segment of the basilar artery's fenestration.^1^ These anomalies present a significant treatment challenge because of their high propensity for rupture^2^ and complex anatomy. Adequate management of BAFAs necessitates a meticulous assessment of the vascular architecture. This assessment should encompass the aneurysm location, the presence of perforating vessels, aneurysm dome insinuation, and the dominance of the fenestration channel, especially for delineating an endovascular treatment plan.^1,3,4^ Furthermore, diligent post-treatment surveillance is crucial for monitoring aneurysm occlusion given the potential for recanalization.^3,5-7^

Adjunctive analyses such as those using computational fluid dynamics (CFD) emerge as potential catalysts for optimizing treatment strategies for complex pathologies like BAFAs, thereby enhancing the assessment of rupture risk and planning the most appropriate technique to be used. Accordingly, this study aims to present and discuss a case of BAFA treated by endovascular means, leveraging pretreatment hemodynamic and anatomic insights obtained through CFD analysis.

## CLINICAL PRESENTATION

**FIGURE 1.**
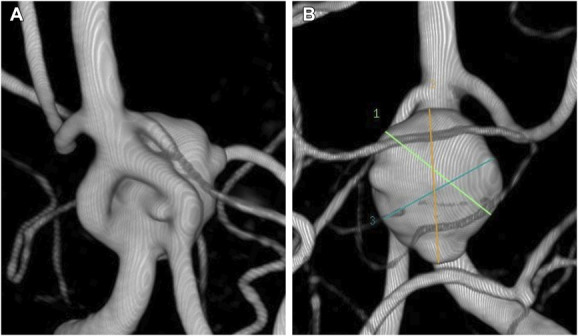
Pretreatment 3-dimensional rotational angiogram images of the basilar artery fenestration aneurysm in **A**, frontal view and **B**, posterior view. The neck opening suggests a more directed inflow toward the right limb of the fenestration, ipsilateral to the dominant vertebral artery.

A 60-year-old woman was incidentally diagnosed with a BAFA during a headache investigation. Digital subtraction angiography (DSA) revealed a 10.6-mm aneurysm with a 4.72-mm neck, located at the lower bifurcation of the BAFA and projecting posteriorly. The neck opening suggested a more directed flow toward the right limb of the fenestration, ipsilateral to the dominant vertebral artery (Figure 1). The patient was on dual antiplatelet therapy (prasugrel 10 mg and aspirin 100 mg) starting 7 days before the first procedure.

Endovascular treatment was initiated under general anesthesia. Right vertebral artery access was achieved using a Neuron Max 088-90 cm (Penumbra) shuttle and a Navien 058 (Medtronic) distal access catheter. Basilar artery catheterization was performed with a Phenom 27 (Medtronic) microcatheter and a Synchro 2-014 (Stryker) microwire. A pipeline embolization device (PED) Shield 3.75 × 18 (Medtronic) was deployed from the basilar artery through the right limb of the fenestration into the right vertebral artery.

**FIGURE 2.**
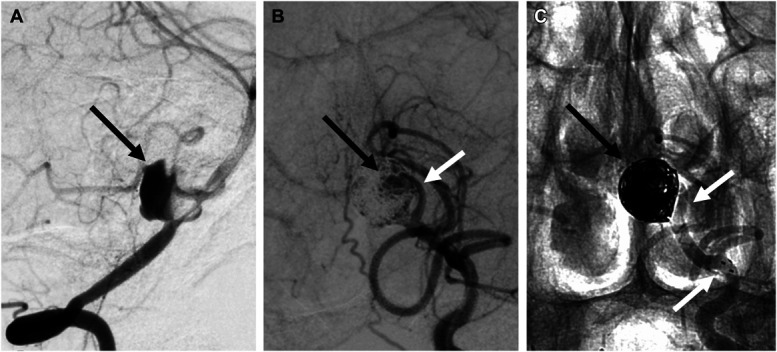
**A**, Image obtained 5 months post–pipeline embolization device shield placement, demonstrating delayed filling of the aneurysm (black arrow) because of flow diversion stent from the basilar artery and right fenestration limb to the right vertebral artery. **B**, Image obtained immediately postreintervention showing residual aneurysm sac after loose coiling (black arrow) and left limb of the basilar fenestration (white arrow). **C**, Coils within the aneurysm sac (black arrow) and left vertebral artery/left fenestration limb Neuroform Atlas stent (white arrows).

**FIGURE 3.**
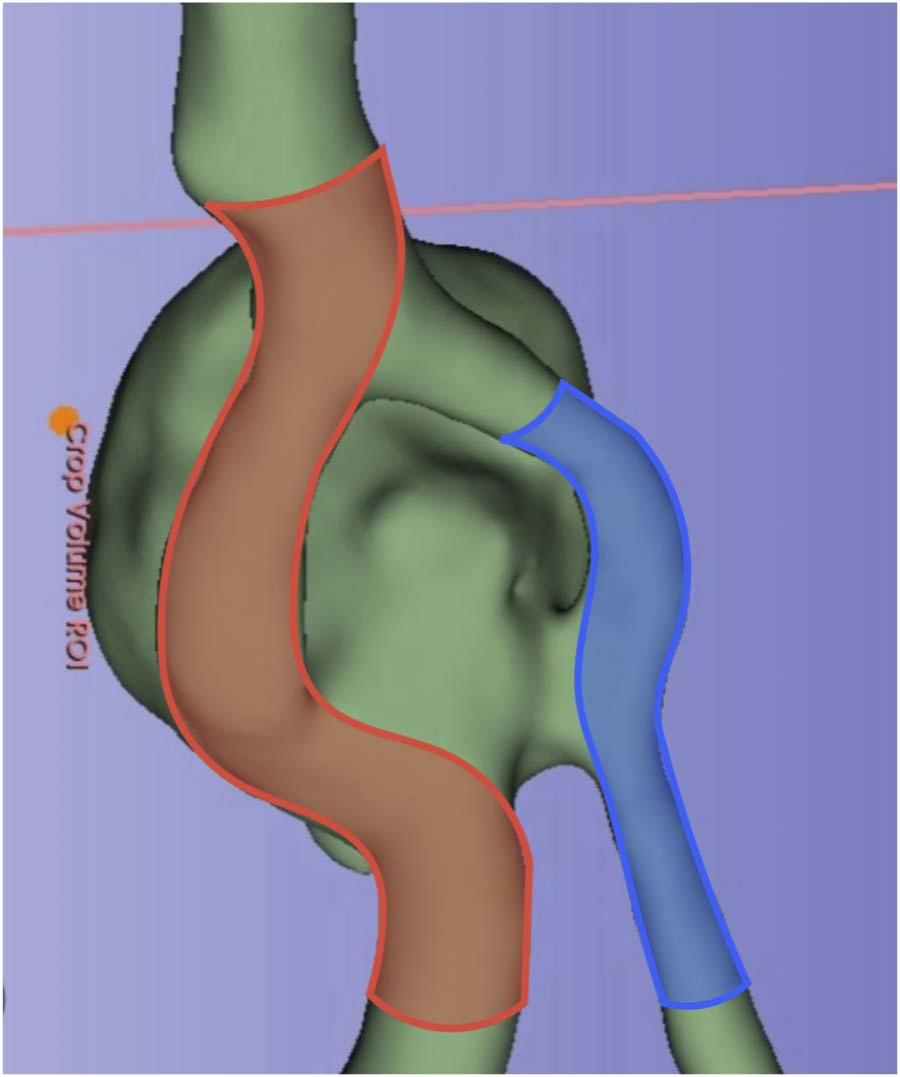
Frontal view of pretreatment aneurysm architecture reconstruction, illustrating the placement of the pipeline embolization device shield (in red) in the basilar artery, extending from the right limb of the fenestration toward the right vertebral artery, and the Neuroform Atlas stent (in blue) from the left vertebral artery to the middle portion of the left limb of the fenestration.

Follow-up angiography at 5 months revealed significant residual opacification of the aneurysm dome (Figure 2). Through the left vertebral artery, a second treatment with stent-assisted loose-packing coil embolization was performed under general anesthesia. The same catheter system was used initially, with the aneurysm catheterized using an Excelsior XT 17 (Stryker) microcatheter and an Avigo-014 (Medtronic) microguidewire. Five Axium coils (Medtronic) were deployed, and a Neuroform Atlas 3.0 × 21 (Stryker) stent was placed from the midleft fenestration to the left vertebral artery, creating a dual approach at the aneurysm neck (Figure 3).

**FIGURE 4.**
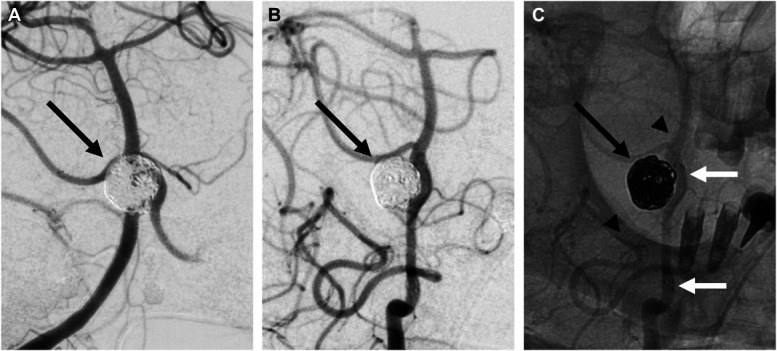
Final outcome post-PED shield and reintervention with stent-assisted loose-packing coil embolization, demonstrating complete occlusion of the basilar fenestration aneurysm (black arrow). **A**, Right vertebral artery injection frontal view. **B**, Left vertebral artery injection digital subtraction angiography oblique view. **C**, Left vertebral artery injection without subtraction showing coils inside of the aneurysm and stent on the left vertebral artery/left limb of the fenestration (white arrows) and plain X-ray image of the PED shield (arrowheads). PED, pipeline embolization device.

The patient continued on dual antiplatelet therapy for 6 months after the second stent, after which prasugrel was switched to clopidogrel 75 mg, while aspirin 100 mg daily was maintained. Follow-up DSA at 6 months postreintervention demonstrated complete aneurysm occlusion (Figure 4).

**FIGURE 5.**
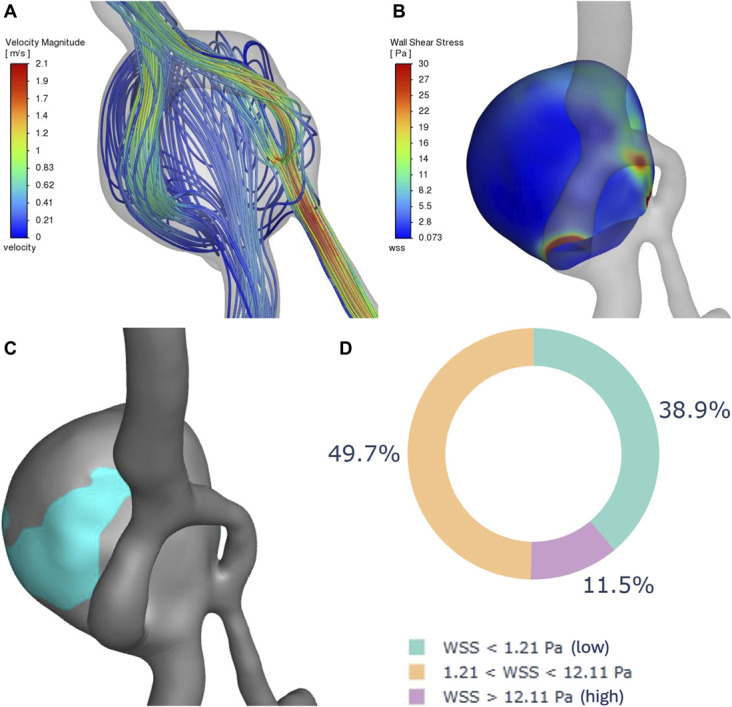
Reconstruction and hemodynamic analyses of the large basilar artery fenestration aneurysm before the initial intervention. **A**, Frontal-oblique view displaying flow patterns and estimated velocities beneath the aneurysm and adjacent arteries. **B**, Frontal-oblique view showing WSS distribution on the aneurysm dome walls. **C**, Frontal-oblique view of the aneurysm surface area exposed to low WSS. **D**, Circular plot analysis presenting the percentages of the aneurysm surface area exposed to low, intermediate, and high WSSs. WSSs, wall shear stresses.

A retrospective CFD analysis of pretreatment hemodynamics and anatomy was conducted. Patient consent was obtained, and pretreatment DSA images were reconstructed using advanced 3-dimensional modeling techniques. Hemodynamic parameters, including flow velocity streamlines, average wall shear stress (WSS), high WSS area, and low WSS area, were analyzed using ANSYS^®^ software (Figure 5). The analysis revealed an average WSS of 2.52 Pa for the aneurysm, with 38.89% of the area exposed to low WSS and 11.5% to high WSS, indicating a high-risk profile.^8-12^ In addition, low WSS was observed in regions corresponding to daughter sacs, another known risk factor for rupture.^13^

## DISCUSSION

BAFAs are rare and pose significant challenges in treatment strategies. A recent systematic review by Essibayi et al^14^ found that most BAFAs present with subarachnoid hemorrhage (74.3%), with only 15% discovered incidentally. The majority of these aneurysms are small (<7 mm) or medium-sized (7-14 mm), projecting anteriorly in 52.6%, posteriorly in 25.9%, laterally in 15.5%, superiorly in 4.5%, and inferiorly in 1.5%.

Endovascular approaches, being less invasive and associated with lower complication rates than surgery, especially in unruptured aneurysms, are the preferred first-line treatment for BAFAs.^14^ Among endovascular options, flow diversion achieves high occlusion rates and preserves perforating vessels but may be less suitable for small-caliber vessels like the basilar artery limbs.^14,15^ By contrast, stent-assisted coiling, while less effective in complete obliteration, offers advantages in reconstructing basilar artery limbs and reducing stroke risk.^14^

The presented case highlights the complex management challenges of treating BAFAs. The incomplete aneurysm occlusion after the initial PED shield intervention led to a reassessment of additional therapeutic strategies. Initially, treatment was based on the impression that the aneurysm's neck was primarily aligned with the dominant right vertebral artery, a finding supported months later by CFD analysis. However, what could not be determined at the time of treatment without CFD was a narrower, high-velocity inflow from the left vertebral artery, filling the aneurysm dome (Video). This resulted in areas of high WSS at the inflow and immediately proximal to it (Figure 5). Furthermore, the CFD analysis indicated a concerning overall rupture risk profile, with a substantial percentage of the aneurysm area exposed to low WSS and a significant area subjected to high WSS.^8-12^

Given the aneurysm's location in the posterior circulation, its size, and its persistence 5 months post-treatment, discontinuing 1 antiplatelet agent to wait for further PED effects was deemed unsafe because of the high rupture risk. The initial treatment with a PED in the dominant fenestration likely failed to achieve complete occlusion because of persistent inflow from the contralateral side. The subsequent intervention—loose-packing coil embolization with a Neuroform Atlas stent—successfully occluded the aneurysm (Figure 4). In retrospect, both procedures could have been combined into a single stage. The critical factor was likely the interface between the aneurysm neck and the inflow zone, which remained patent because of significant inflow from the contralateral nondominant fenestration. CFD performed before treatment could have identified this hemodynamic issue, suggesting a combined approach to both fenestration limbs, potentially achieving complete occlusion in 1 stage and highlighting the role of CFD in optimizing planning for complex cases.

The loose-packing strategy, combined with stenting of the nondominant fenestration and the previously deployed PED in the dominant fenestration, was chosen to minimize brainstem compression while promoting aneurysm thrombosis. Loose-packing with PED has been effective in large, wide-necked, or complex aneurysms with high rupture risk^16-18^ although its use with conventional stents has shown variable outcomes when accounting for confounding factors.^19^

Finally, as our understanding of WSS on aneurysm walls and the impact of competitive inflow zones in fenestrated anatomies evolves, this case highlights the value of incorporating advanced imaging techniques like CFD into the therapeutic decision-making process for complex cerebrovascular cases.

### Limitations

While this study reports the potential benefits of complementary CFD analysis, future research involving patient cohorts is necessary to further understand the limitations and practical applications of this tool in managing similar complex cases.

## CONCLUSION

The management of BAFAs is challenging because of the unique anatomic variations in each case. Individualized treatment strategies, guided by objective data, are essential for optimizing patient outcomes. In this case, we analyzed the BAFA's morphology and pretreatment hemodynamics using CFD, underscoring its potential as a valuable adjunct and complementary tool in the therapeutic planning of complex cerebrovascular pathologies.
